# Overnight staffing in Canadian neonatal and pediatric intensive care units

**DOI:** 10.3389/fped.2023.1271730

**Published:** 2023-11-01

**Authors:** Christina Maratta, Kristen Hutchison, Jessica Nicoll, Sean M. Bagshaw, John Granton, Haresh Kirpalani, Henry Thomas Stelfox, Niall Ferguson, Deborah Cook, Christopher S. Parshuram, Gregory P. Moore

**Affiliations:** ^1^ Inter-Departmental Division of Critical Care Medicine, University of Toronto, Toronto, ON, Canada; ^2^ Department of Critical Care Medicine, Hospital for Sick Children, Toronto, ON, Canada; ^3^ Department of Paediatrics, University of Toronto, Toronto, ON, Canada; ^4^ Institute of Health Policy, Management and Evaluation, University of Toronto, Toronto, ON, Canada; ^5^ Child Health and Evaluative Sciences, SickKids Research Institute, Toronto, ON, Canada; ^6^ Centre for Safety Research, Sick Kids Research Institute, Toronto, ON, Canada; ^7^ Department of Critical Care Medicine, Faculty of Medicine and Dentistry, University of Alberta, Edmonton, AB, Canada; ^8^ Department of Paediatrics, University of Pennsylvania, Philadelphia, PA, United States; ^9^ Department of Critical Care Medicine and O’Brien Institute for Public Health, University of Calgary & Alberta Health Services, Calgary, AB, Canada; ^10^ Department of Medicine and Physiology, University of Toronto, Toronto, ON, Canada; ^11^ Department of Medicine and Clinical Epidemiology & Biostatistics, McMaster University, Hamilton, ON, Canada; ^12^ Division of Critical Care Medicine, McMaster University, Hamilton, ON, Canada; ^13^ Division of Neonatology, Children’s Hospital of Eastern Ontario, Ottawa, ON, Canada; ^14^ Division of Newborn Care, The Ottawa Hospital, Ottawa, ON, Canada; ^15^ Faculty of Medicine, University of Ottawa, Ottawa, ON, Canada; ^16^ Clinical Research Unit, Research Institute, Children’s Hospital of Eastern Ontario, Ottawa, ON, Canada

**Keywords:** PICU (pediatric intensive care unit), NICU (neonatal intensive care unit), overnight, staffing, pediatric critical care, neonatal critical care

## Abstract

**Aim:**

Infants and children who require specialized medical attention are admitted to neonatal and pediatric intensive care units (ICUs) for continuous and closely supervised care. Overnight in-house physician coverage is frequently considered the ideal staffing model. It remains unclear how often this is achieved in both pediatric and neonatal ICUs in Canada. The aim of this study is to describe overnight in-house physician staffing in Canadian pediatric and level-3 neonatal ICUs (NICUs) in the pre-COVID-19 era.

**Methods:**

A national cross-sectional survey was conducted in 34 NICUs and 19 pediatric ICUs (PICUs). ICU directors or their delegates completed a 29-question survey describing overnight staffing by resident physicians, fellow physicians, nurse practitioners, and attending physicians. A comparative analysis was conducted between ICUs with and without in-house physicians.

**Results:**

We obtained responses from all 34 NICUs and 19 PICUs included in this study. A total of 44 ICUs (83%) with in-house overnight physician coverage provided advanced technologies, such as extracorporeal life support, and included all ICUs that catered to patients with cardiac, transplant, or trauma conditions. Residents provided the majority of overnight coverage, followed by the Critical Care Medicine fellows. An attending physician was in-house overnight in eight (15%) out of the 53 ICUs, seven of which were NICUs. Residents participating in rotations in the ICU would often have rotation durations of less than 6 weeks and were often responsible for providing care during shifts lasting 20–24 h.

**Conclusion:**

Most PICUs and level-3 NICUs in Canada have a dedicated in-house physician overnight. These physicians are mainly residents or fellows, but a notable variation exists in this arrangement. The potential effects on patient outcomes, resident learning, and physician satisfaction remain unclear and warrant further investigation.

## Introduction

Intensive care units (ICUs) provide continuous care to critically ill children and newborns. The quality of care significantly affects the survival rates and the neurocognitive outcomes of approximately 95% of children who survive an ICU admission in Canada each year (1–4). While multi-disciplinary ICU care led by intensivists may improve outcomes (5, 6), it is unclear whether increasing ICU physician engagement by mandating overnight presence of fully certified specialists is beneficial (7–9). Moreover, implementing this strategy is complicated by workforce and funding limitations. The utilization of trainees and other caregivers for staffing purposes ensures the continuous presence of in-house physicians, allowing for the timely addressing of various clinical, educational, and stakeholder-related needs.

We previously described physician staffing in Canadian pediatric ICUs (PICUs) in 2006, where it was observed that only one PICU mandated in-house call for attending intensivists (10). According to the Canadian Neonatal Network, the presence of senior in-house physicians in neonatal ICUs (NICUs) has been associated with a decreased mortality rate among newborns (11). In the intervening decade, several changes have occurred. These include new regulations on resident work hours, expectations of physician engagement, and improved outcomes following NICU and PICU admission (12–14). The objective of this study is to describe overnight staffing practices in Canadian NICUs (level 3) and PICUs.

## Materials and methods

We conducted a cross-sectional survey of directors of PICUs and NICUs across Canada. Eligible ICUs were level-3 NICUs or PICUs (15). Level-3 NICUs must provide acute critical care to infants (16). We defined ICU as an area that routinely provided invasive mechanical ventilation for >48 h, and where other advanced life support therapies may be provided.

### Survey design

The design of this questionnaire was informed by the results of our 2006 survey on staffing in adult ICU and PICU (10). All respondents were asked 29 items describing the ICU's capacity, technology, staffing, and duty duration; the neonatal questionnaire included one additional item related to delivery room coverage. NICU and PICU directors were also asked to describe their typical staffing, as well as the staffing that was actually observed during the month of February 2017. Institutional review board approval was obtained at the Hospital for Sick Children in Toronto prior to study initiation (approval no. 1000008569).

### Survey administration

All PICUs and level-3 NICUs were identified from a report by the Canadian Institutes of Health Information (CIHI) and the Canadian Neonatal Network database. The ICU director was contacted to confirm the eligibility of the ICU and was invited to participate in the study. Surveys were sent by mail or email to be completed by the director or a delegate. Surveys were returned by mail, fax, or email to the study team between March and August 2017. A maximum of four reminders were sent by email and/or telephone.

### Outcomes

The primary outcome was overnight physician coverage, defined as the presence or absence of a physician who was physically present in the hospital to provide care in the ICU overnight. Descriptions of in-house physician coverage included seniority, overnight shift duration, clinical responsibilities outside the ICU, and the availability of advance practice nurses or physician assistants. Descriptions of each ICU included the ICU type and size and the available ICU technologies. The assessment of ICU size encompassed the evaluation of the number of ministry-funded beds, the number of ventilated beds, and the annual count of patient admissions.

### Data management and analyses

Surveys that lacked complete primary outcome data required ICU directors to provide clarification for missing survey responses. Response rates were calculated as the proportion of ICUs to which questionnaires were sent overall, as well as separately for NICUs and PICUs, respectively. Data were transcribed into a bespoke database (Oracle Corporation, Austin, TX, USA). Descriptive statistics were used to describe ICU characteristics, and by ICUs with and without overnight in-house coverage. The continuous data were described as median with interquartile range (IQR), while proportions were described as the number and percentage using SAS v9.4 statistical software (SAS N.C, USA).

## Results

We identified 53 eligible pediatric and neonatal ICUs, and all units opted to participate in the study. The directors of each ICU confirmed their eligibility and agreed to participate. We received responses from all 19 PICUs and 34 NICUs, resulting in an overall response rate of 100%.

The 53 ICUs provided care for 35,457 patient admissions in 2016, ranging from 200 to 1,500 admissions annually. High frequency oscillatory ventilation (HFOV) and inhaled nitric oxide (iNO) were available in 46 (87%) ICUs. Continuous renal replacement therapy (CRRT) was available in 16 (30%) ICUs, and extracorporeal membrane oxygenation (ECMO) was available in 12 (23%) ICUs (Table 1). An ICU-based Rapid Response Team was present in association with 23% of ICUs.

**Table 1:** Demographic characteristics of participating neonatal and pediatric ICUs.

Characteristic	All, *n* = 53	NICU, *n* = 34	PICU, *n* = 19
ICU beds			
Maximum, (IQR)	20 (12–34)	25 (20–48)	12 (6–18)
Ventilated, (IQR)	N/A	N/A	12 (6–16)
Admissions in 2016, (IQR)	700 (500–900)	675 (500–900)	702 (435–833)
Routinely admitted patients, *n* (%)			
Cardiac	17 (32)	10 (29)	7 (37)
Transplantation	7 (13)	0 (0)	7 (37)
Trauma	16 (30)	0 (0)	16 (84)
ICU technologies available, *n* (%)			
CRRT	16 (30)	2 (6)	14 (74)
ECMO	12 (23)	1 (3)	11 (58)
HFOV	46 (87)	30 (88)	16 (84)
Nitric oxide	46 (87)	29 (85)	17 (89)
SLED	5 (9)	0 (0)	5 (26)
VAD	5 (9)	0 (0)	5 (26)
Rapid response team, *n* (%)	12 (23)	0 (0)	12 (63)
Any overnight in-house physician, *n* (%)	44 (83)	28 (82)	16 (84)
Attending in-house physician overnight, *n* (%)	8 (15)	7 (21)	1 (5)
Nurse practitioner available, *n* (%)	24 (45)	18 (53)	6 (32)
Overnight	7 (13)	7 (21)	0 (0)
Physician assistant available, *n* (%)	6 (11)	4 (12)	2 (11)
Overnight	5 (9)	4 (12)	1 (5)

SLED, sustained low-efficiency dialysis.

Continuous variables are presented as medians and interquartile ranges (IQR).

The 34 (100%) NICUs had 1,141 beds with a median (IQR) of 25 (20–48) ventilated beds per ICU. In 2016, there was a total of 22,039 admissions, with a median (IQR) of 675 (500–900) admissions per ICU. HFOV was available in 30 (88%) NICUs, and iNO was available in 29 (85%) NICUs. CRRT was used in two (6%) NICUs, and one (3%) had ECMO. Nurse practitioners were employed in 18 (53%) NICUs, of which seven (21%) employed nurse practitioners overnight.

The 19 (100%) PICUs had 246 beds, including 232 beds that were allocated for mechanical ventilation. Each ICU had a median (IQR) of 12 (6–18) beds. In 2016, PICUs had 13,418 admissions, with a median (IQR) of 702 (435–833) admissions per ICU. Seven PICUs routinely admitted cardiac patients, and two were specialized Pediatric Cardiac Critical Care Units. HFOV was available in 16 (84%) PICUs, CRRT was available in 14 (74%) PICUs, ECMO was used in 11 (58%) PICUs, and five (26%) PICUs had ventricular assist devices (VAD). Six (32%) PICUs had nurse practitioners, none of which had nurse practitioners overnight. Twelve (63%) PICUs had a Rapid Response Team.

One or more overnight in-house physicians were present in 44 (83%) ICUs: 28 (82%) NICUs and 16 (84%) PICUs had overnight in-house physician coverage. ICUs with in-house physicians overnight had more ventilated beds and more admissions and offered more advanced therapeutic technologies. All ICUs providing specialized care in cardiothoracic surgery, transplant, or trauma had in-house overnight physicians (Table 2). Eight (15%) of the sites evaluated, which comprised one (5%) PICU and seven (21%) NICUs, had an attending in-house physician present overnight.

**Table 2:** Comparison of PICUs and NICUs with and without in-house overnight physicians.

Characteristic	NICU		PICU	
	With in-house physician, *n* = 28	Without in-house physician, *n* = 6	With in-house physician, *n* = 16	Without in-house physician, *n* = 3
ICU beds				
Maximum, median (IQR)	34 (21–51)	19 (12–20)	12 (9–19)	5 (4–6)
Ventilated, median (IQR)	N/A	N/A	12 (9–17)	5 (4–6)
Unit admissions in 2016, median (IQR)	750 (500–950)	560 (500–600)	750 (539.5–866.5)	380 (300–700)
All admissions in 2016	19,479	2,560	12,038	1,380
Routinely admitted patients, *n* (%)				
Cardiac	10 (36)	0 (0)	7 (44)	0 (0)
Transplantation	0 (0)	0 (0)	7 (44)	0 (0)
Trauma	0 (0)	0 (0)	13 (81)	3 (100)
Available technologies, *n* (%)				
CRRT	2 (7)	0 (0)	14 (88)	0 (0)
ECMO	1 (4)	0 (0)	11 (69)	0 (0)
HFOV	27 (96)	3 (50)	15 (94)	1 (33)
Nitric oxide	27 (96)	2 (33)	14 (88)	3 (100)
SLED	0 (0)	0 (0)	5 (31)	0 (0)
VAD	0 (0)	0 (0)	5 (31)	0 (0)
Rapid response team, *n* (%)	0 (0)	0 (0)	12 (75)	0 (0)
Nurse practitioner available, *n* (%)	17 (61)	1 (17)	6 (38)	0 (0)
Overnight	7 (25)	0 (0)	0 (0)	0 (0)
Physician Assistant available, *n* (%)	4 (14)	0 (0)	2 (13)	0 (0)
Overnight	4 (14)	0 (0)	1 (6)	0 (0)

Continuous variables are presented as medians and interquartile ranges (IQR).

Most of the NICUs and PICUs were staffed with year 1 (R1) residents (29/53, 55%) and year 2–5 (R2–R5) residents (41/53, 77%) in February 2017 (Table 3). Half of the ICUs had dedicated Critical Care Medicine (CCM) fellows (27/53, 51%). Overnight, NICUs and PICUs were predominantly staffed by a mix of R1s, R2–R5s and CCM fellows. In the NICUs, the most senior in-house physician was often one of the following: an R2–R5 (18%), a CCM fellow (29%), a clinical associate (15%), or an attending physician (21%).

**Table 3:** Description of daytime and overnight in-house staffing of PICUs and NICUs in February 2017.

NICU (*n* = 34)	Intern (R1) ICUs	Resident (R2–R5) ICUs	CCM fellow ICUs	Clinical scholar ICUs	Clinical associate ICUs	Attending physician ICUs
Usually work in ICU, *n* (%)	22 (65)	27 (79)	19 (56)	3 (9)	11 (32)	N/A
Worked in ICU February 2017, *n* (%)	22 (65)	26 (76)	19 (56)	2 (6)	11 (32)	N/A
Worked overnight in-house in ICU in February 2017, *n* (%)	18 (53)	25 (74)	18 (53)	1 (3)	8 (24)	7 (21)
Most senior in-house physician overnight, *n* (%)	5 (15)	6 (18)	10 (29)	1 (9)	5 (15)	7 (21)
Duty length^a^, *n* (%) (h)						
<8	0 (0)	0 (0)	0 (0)	0 (0)	0 (0)	0 (0)
8 to <12	3 (9)	4 (12)	2 (6)	0 (0)	3 (9)	2 (6)
12 to <16	4 (12)	5 (15)	5 (15)	0 (0)	6 (18)	4 (12)
16 to <20	1 (3)	4 (12)	4 (12)	0 (0)	1 (3)	0 (0)
20 to <25	8 (24)	12 (35)	11 (32)	1 (3)	5 (15)	4 (12)
25 to <30	3 (9)	2 (6)	2 (6)	0 (0)	2 (6)	2 (6)
30 to <36	0 (0)	0 (0)	0 (0)	0 (0)	0 (0)	2 (6)
≥36	0 (0)	1 (3)	1 (3)	0 (0)	0 (0)	2 (6)
Speciality, *n* (%)						
Anesthesia	6 (18)	10 (29)	5 (15)	0 (0)	4 (12)	3 (9)
Cardiology	0 (0)	0 (0)	0 (0)	0 (0)	0 (0)	0 (0)
Emergency medicine	0 (0)	1 (3)	1 (3)	0 (0)	1 (3)	0 (0)
Family medicine	5 (15)	7 (21)	3 (9)	0 (0)	5 (15)	3 (9)
Internal medicine	0 (0)	0 (0)	0 (0)	0 (0)	0 (0)	0 (0)
Nephrology	0 (0)	0 (0)	0 (0)	0 (0)	0 (0)	0 (0)
Pediatrics	20 (6)	24 (71)	19 (56)	2 (6)	11 (32)	7 (21)
Respirology	0 (0)	0 (0)	1 (3)	0 (0)	1 (3)	1 (3)
Surgery	0 (0)	0 (0)	1 (3)	1 (3)	0 (0)	0 (0)
Other	10 (29)	10 (29)	8 (24)	1 (3)	2 (6)	3 (9)
ICU rotation duration, *n* (%)						
<4 weeks	10 (29)	17 (50)	5 (15)	1 (3)	N/A	N/A
4–6 weeks	6 (18)	9 (26)	2 (6)	0 (0)		
7–9 weeks	4 (12)	0 (0)	0 (0)	0 (0)		
2–3 months	0 (0)	0 (0)	1 (3)	0 (0)		
>3 months	0 (0)	0 (0)	10 (29)	1 (3)		
PICU (*n* = 19)	Intern (R1) ICUs	Resident (R2-5) ICUs	CCM fellow ICUs	Clinical scholar ICUs	Clinical associate ICUs	Attending physician ICUs
Usually work in ICU, *n* (%)	8 (42)	15 (79)	9 (47)	3 (16)	6 (32)	N/A
Worked in ICU February 2017, *n* (%)	7 (37)	15 (79)	8 (42)	4 (21)	6 (32)	N/A
Worked overnight in-house in ICU in February 2017, *n* (%)	5 (26)	14 (74)	9 (47)	3 (16)	6 (32)	1 (5)
Most senior in-house physician overnight, *n* (%)	0 (0)	7 (37)	2 (11)	1 (5)	5 (26)	1 (5)
Duty length^a^, *n* (%) (h)						
<8	0 (0)	0 (0)	0 (0)	0 (0)	0 (0)	0 (0)
8 to <12	1 (5)	1 (5)	0 (0)	0 (0)	0 (0)	0 (0)
12 to <16	3 (16)	4 (21)	2 (11)	1 (5)	2 (11)	0 (0)
16 to <20	1 (5)	3 (16)	2 (11)	0 (0)	1 (5)	0 (0)
20 to <25	2 (11)	8 (42)	5 (26)	1 (5)	4 (21)	1 (5)
25 to <30	0 (0)	1 (5)	2 (11)	1 (5)	1 (5)	0 (0)
30 to <36	0 (0)	0 (0)	0 (0)	0 (0)	0 (0)	0 (0)
≥36	0 (0)	1 (5)	0 (0)	0 (0)	0 (0)	1 (5)
Speciality, *n* (%)						
Anesthesia	1 (5)	8 (42)	7 (37)	3 (16)	4 (21)	1 (5)
Cardiology	0 (0)	1 (5)	2 (11)	1 (5)	1 (5)	0 (0)
Emergency medicine	0 (0)	10 (53)	6 (32)	2 (11)	4 (21)	1 (5)
Family medicine	2 (11)	4 (21)	0 (0)	1 (5)	1 (5)	0 (0)
Internal medicine	0 (0)	0 (0)	0 (0)	0 (0)	0 (0)	0 (0)
Nephrology	0 (0)	0 (0)	0 (0)	0 (0)	0 (0)	0 (0)
Pediatrics	0 (0)	14 (74)	8 (42)	4 (21)	6 (32)	1 (5)
Respirology	4 (21)	2 (11)	2 (11)	0 (0)	1 (5)	0 (0)
Surgery	0 (0)	1 (5)	1 (5)	0 (0)	0 (0)	0 (0)
Other	0 (0)	1 (5)	0 (0)	0 (0)	0 (0)	0 (0)
ICU rotation duration, *n* (%)						
<4 weeks	5 (26)	9 (47)	0 (0)	0 (0)	N/A	N/A
4–6 weeks	2 (11)	4 (21)	0 (0)	0 (0)		
7–9 weeks	0 (0)	1 (5)	1 (5)	0 (0)		
2–3 months	0 (0)	0 (0)	1 (5)	1 (5)		
>3 months	0 (0)	0 (0)	7 (27)	1 (5)		

**Interns** [R1 resident or Post-Graduate Year 1 (PGY1)]: First year medical resident. **Resident:** Trainee in post-graduate specialty training with focus that is not critical care medicine. (e.g., internal medicine, cardiology, respirology, anesthesia, pediatrics). R2–R5 indicated post-graduate years 2 through 5. **CCM (Critical Care Medicine) fellow:** Trainee in a program designed specifically to become a critical care physician. Note some “residents” may fall into this category that is intended to reflect critical care trainees. CCM fellow is meant to indicate NICU fellow for the NICU and PICU fellow for the PICU. **Clinical Scholar:** A senior trainee who has completed clinical training and who functions as a junior staff physician, but is responsible to a staff physician, and is undertaking additional advanced training (research or other). **Clinical Associate:** A physician who is not in training, is not a staff physician, and reports to the staff physician regarding ICU patients. **Attending Physician:** The critical care physician who assumes responsibility for patients while in the ICU. In some units, these physicians may be called intensivists, ICU physician, ICU Coordinator or “the P/NICU staff physician”. In the NICU, this physician may be called Staff Neonatologist.

^a^ Duty length for only those physician groups that worked overnight duty in February 2017. Duty lengths do not need to amount to the sum of the column as trainees may have variable duty durations within a given period.

The ICUs were predominantly staffed by pediatric trainees or attending physicians. However, trainees from many medical specialties, including Anesthesia, Family Medicine, Emergency Medicine, and Cardiology, also participated in rotations inside both NICUs and PICUs. Most trainees provided care for periods of 6 weeks or less, except for CCM fellows working in the PICU who often worked for periods >3 months. A duty duration of 20–24 h was found to be prevalent among all types of physicians providing care, especially among R1–R5 residents, CCM fellows, and attending physicians (Table 3).

## Discussion

This national cross-sectional survey of NICU and PICU staffing describes the ICU capacity and staffing patterns for neonates and children across Canada for the contemporary period prior to the COVID-19 pandemic. Eighty-three percent of all the 53 identified ICUs had a dedicated in-house physician staffing during overnight hours. This is higher than that of adult ICUs in Canada (17). We found that the NICUs and PICUs with physicians (residents, fellows, or attending physicians) in-house overnight were primarily located in either freestanding children's hospitals or in regional academic centers. In addition, these units were equipped with advanced technologies such as ECMO, VAD, and CRRT. Where in-house overnight physician staffing was available, it tended to be in larger ICUs with greater medical complexity or where advanced therapies would be administered.

Physicians working in-house overnight were predominantly pediatric residents and CCM fellows. Attending physicians were rarely mandated to be in-house overnight in NICUs, and were only required to be in-house overnight in a single PICU. There were differences between practices described in the United States, where approximately half of NICUs and PICUs have attending physicians in-house overnight. However, comparisons are limited due to significant differences in staffing task force expectations and funding (18, 19). Similar severity-adjusted mortality rates in the United States and Canada may indicate support for the staffing model with overnight trainee coverage that we report. While some studies conducted in the United States report a decrease in mortality rates in ICUs with in-house physicians present overnight, this effect is attenuated or absent in ICUs with training programs (18, 20, 21).

From an educational standpoint, staffing decisions also reflect a balance between competency for overnight coverage and progressive autonomy for trainees. The discretionary presence of the attending physician, together with the availability of in-house nurse practitioners and other trainees present overnight, may mitigate potential drawbacks from a trainee's perspective. North American trainees reported that the requirement of having an in-house attending coverage in the PICU overnight may be beneficial for their educational experience. However, there are some trainees who have expressed concerns regarding their ability to move to independent practice once their training period with in-house attending coverage ended (22). The increase in the number of overnight attending neonatologist in-house coverage in the United States has also resulted in concerns regarding the competencies of future neonatologists (23) and may serve to fuel the future “need” for in-house attending ICU physicians in NICUs and PICUs.

We found that current staffing patterns in PICUs resembled those from the results of our survey in 2006 (10, 24). In our previous study, 16 out of 18 PICUs had reported the presence of a physician on duty overnight, and one had an in-house attending physician overnight. Findings of the status quo may reflect financial implications of mandated in-house model for the healthcare system or unresolved workforce limitations. Alternatively, the clinical needs of critically ill neonates and children may simply be unchanged.

In contrast, the current NICU data suggest that overnight attending coverage may have changed over time. In 1996, a survey of 17 Canadian level-3 NICUs reported that one NICU had an in-house attending physician overnight (11) compared with the seven (21%) with an in-house attending physician overnight of the 34 NICUs we found in the current data. This finding may reflect a smaller number of NICUs with CCM fellows compared with PICUs, differences in the sampled NICUs, or other factors, such as increasing ICU care delivered to patients at lower gestational ages.

The strengths of this study include a 100% response rate, national representation, a comprehensive description of ICUs, and a direct comparison afforded by a similar prior survey. There are several limitations to this study. First, the survey did not capture the duration or frequency that attending physicians were in-house during discretionary home-call. It is probable that these attending physicians will be present at the patient’s bedside during the admission and during the management of a deteriorating critically ill neonate or child. It is possible that if actual bedside presence of attending physicians with discretionary in-house presence were assessed, an appreciable difference in patient-level outcomes between mandated and discretionary overnight presence would be unlikely. Second, the questionnaire did not assess patient outcomes such as survival, length of stay, safety events, or long-term functional outcomes. Third, the learning environment for residents and fellows was not assessed nor was their perception of readiness for independent practice. Fourth, these data reflect the practices of Canadian NICUs and PICUs in 2017. While dating a few years back, they do provide insight into the practice and training landscape prior to the COVID-19 pandemic. However, it is important to note that the survey was completed prior to the pandemic, and therefore, there might have been some changes to staffing models and overnight presence. Although significant changes due to the pandemic are unlikely in the pediatric setting, these potential modifications will be assessed in future surveys. Finally, the professional satisfaction and burnout levels of attending physicians, fellows, and residents were not captured in the survey. These issues need to be further explored.

## Conclusion

We conducted a national cross-sectional survey of 34 level-3 NICUs and 19 PICUs in Canada. We documented that 83% of the ICUs have overnight in-house physicians, with these physicians predominantly being pediatric residents and fellows who work in duty periods of 24 h. Notable variation was observed among ICUs. A minority (15%) of ICUs had a constant presence of in-house attending physicians, which is consistent with the findings of our previous national survey of PICU in-house attending coverage. However, it is worth noting that the presence of attending physicians overnight may have increased in NICUs across Canada (11). Further investigation is warranted to examine the effect of the differences in physician staffing on patient care processes and outcomes, as well as trainee education and readiness for independent practice.

## Data Availability

The raw data supporting the conclusions of this article will be made available by the authors, without undue reservation.
